# EphA‐Mediated Regulation of Stomatin Expression in Prostate Cancer Cells

**DOI:** 10.1002/cam4.70276

**Published:** 2024-10-08

**Authors:** Masanari Nishida, Akira Sato, Akio Shimizu, Nor Idayu A. Rahman, Akinori Wada, Susumu Kageyama, Hisakazu Ogita

**Affiliations:** ^1^ Division of Molecular Medical Biochemistry, Department of Biochemistry and Molecular Biology Shiga University of Medical Science Otsu Japan; ^2^ Department of Urology Shiga University of Medical Science Otsu Japan

**Keywords:** gene expression regulation, molecular biology, prostate cancer, signal transduction

## Abstract

**Background and Aims:**

Tumor growth and progression are affected by interactions between tumor cells and stromal cells within the tumor microenvironment. We previously showed that the expression of an integral membrane protein, called stomatin, was increased in cancer cells following their association with stromal cells. Additionally, stomatin impaired the Akt signaling pathway to suppress tumor growth. However, it remains unclear how stomatin expression is regulated. To explore this, we examined the cell surface molecules that can transduce the intercellular communication signals between cancer cells and stromal cells.

**Results:**

Among these molecules, EphA3 and EphA7 receptors and their ligand ephrin‐A5 were found to be expressed in prostate cancer cells, but not in prostate stromal cells. Cell‐to‐cell contact of prostate cancer cells through the EphA–ephrin‐A interaction suppressed stomatin expression, while knockdown of EphA3/7 or ephrin‐A5 increased stomatin expression. This increase contributed to an inhibition of prostate cancer cell proliferation. Intracellularly, the binding of ephrin‐A to EphA attenuated extracellular signaling‐regulated kinase (ERK) activation that promoted stomatin expression. Furthermore, ELK1 and ELK4, which are Ets family transcription factors phosphorylated by ERK, were involved in the induction of stomatin expression. We also found that higher Gleason score prostate cancer tissue samples had increased activation of EphA, while the stomatin expression and activated ERK and ELK levels were all low. In the mouse xenograft tumor samples generated by implantation of prostate cancer cells, EphA3 phosphorylation was attenuated and the ERK–ELK signaling and stomatin expression were enhanced in the area where stromal cells infiltrated the tumor.

**Conclusion:**

The EphA‐mediated signaling suppresses the ERK–ELK pathway, leading to the reduction of stomatin expression that affects prostate cancer malignancy.

## Graphical abstract

Stomatin expression, which was positively regulated by the ERK‐ELK signaling axis, was suppressed by EphA–ephrin‐A interaction between neighboring prostate cancer cells. Because stomatin strongly inhibited tumor progression, this EphA‐mediated suppression of stomatin contributed to tumor malignancy.

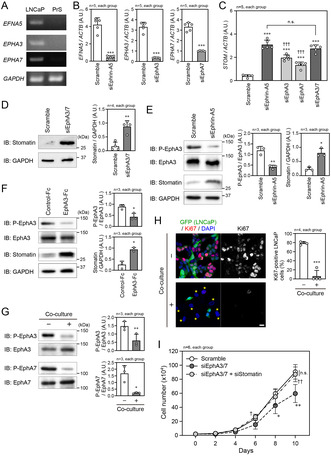

## 1 Introduction

Tumor growth and progression involve multiple complex processes mediated by interactions between cancer cells and their surrounding stroma. The stroma includes diverse cell types, such as immune cells and fibroblasts, and acellular components, such as the extracellular matrix, forming the tumor microenvironment [1]. The outcomes of the tumor cell–stroma interaction lead to positive or negative regulation of tumor progression, depending on the tumor type, developmental stage, and location [2]. Within the tumor microenvironment, direct cell‐to‐cell interactions play important roles in regulating tumor behaviors.

Previously, we used an *in vitro* co‐culture system of LNCaP prostate cancer cells with prostate stromal (PrS) cells to investigate the genes that were involved in regulating tumor behavior via cell‐to‐cell contact. We identified 30 genes that showed significantly increased expression levels in cancer cells after they were associated with PrS cells. The *STOM* gene was among these, which encodes a 31 kDa integral membrane protein enriched in erythrocytes and is called stomatin. This protein possesses an intramembrane domain, two palmitoylated cysteines, and a coiled‐coil domain, in addition to the conserved Stomatin, Prohibitin, Flotillin, and HflK/C (SPFH) domain [3, 4]. Stomatin mainly localizes on lipid rafts to regulate the activities of several channels and transporters [5, 6, 7]. We have recently revealed that stomatin displays strong anti‐tumor activity by inhibiting cell proliferation and inducing apoptosis in cancer cells. Mechanistically, stomatin bound to phosphoinositide‐dependent protein kinase 1 (PDPK1), a major activator of Akt, and has impaired PDPK1 protein stability. Therefore, stomatin decreased the PDPK1 protein level and thereby attenuated the Akt‐mediated signaling pathways to act as a tumor suppressor [8]. However, it remains unclear how stomatin expression is up‐regulated by the heterogeneous cell‐to‐cell contact between prostate cancer cells and PrS cells.

To examine the cell‐to‐cell contact‐dependent regulation of stomatin expression, we performed a data search using Harmonizome [9], which suggested EphA3, EphA7, and ephrin‐A5 in LNCaP cells as top‐ranked candidates. EphA is a receptor tyrosine kinase, while ephrin‐A is its corresponding ligand. Ephrin‐A is tethered on the plasma membrane through a glycosylphosphatidylinositol anchor and lacks a cytoplasmic domain [10]. The cell‐to‐cell communication between LNCaP cells via these molecules suppressed stomatin expression. EphA‐mediated intracellular signaling attenuated extracellular signaling‐regulated kinase (ERK) activation that promoted stomatin expression. Downstream of ERK, Ets family transcription factors ELK1 and ELK4 were also involved in the regulation of stomatin expression. Consistent with these results, immunohistochemistry (IHC) assays suggested that prostate cancer samples with a higher Gleason score (GS) show high EphA3 phosphorylation, low ERK1/2 and ELK1 phosphorylation, and low stomatin expression levels. Prostate cancer samples with a lower GS displayed the opposite trends, indicating that EphA3 signaling and stomatin expression contribute to prostate cancer malignancy. Finally, in the mouse xenograft samples generated by implantation of LNCaP cells, EphA3 phosphorylation observed in the tumor was attenuated by infiltration of stromal cells into the tumor, and this infiltration conversely activated the ERK–ELK signaling, resulting in enhancement of stomatin expression.

## 2 Materials and Methods

### 2.1 Cell Culture

LNCaP cells (RRID: CVCL_0395) and PC3M cells (RRID: CVCL_9555) were purchased from Dainippon Sumitomo Pharma (Osaka, Japan) and cultured in RPMI 1640 medium (Nacalai Tesque, Kyoto, Japan) supplemented with 10% fetal bovine serum (FBS; Biosera, Cholet, France), 20 mmol/L L‐glutamine (Nacalai Tesque), and 100 U/mL penicillin–streptomycin (Nacalai Tesque). Human PrS cells were purchased from Lonza (Basel, Switzerland) and cultured in Stromal cell basal medium supplemented with growth factors (Lonza). Both types of cells were cultured at 37°C in a humidified atmosphere of 5% CO_2_ and 95% air.

### 2.2 Reagents

PD98059 was obtained from Fujifilm Wako (Osaka, Japan). BCI was obtained from MedChemExpress (Monmouth Junction, NJ, USA). LNCaP cells were treated with 5 μmol/L PD98059 or BCI for 24 h. Recombinant human EphA3‐Fc was obtained from R&D systems (Minneapolis, MN, USA). LNCaP cells were treated with 250 ng/mL EphA3‐Fc for 24 h.

### 2.3 Mammalian Expression Plasmids and Introduction of Plasmids Into Cells

To construct pFLAG‐CMV5a‐EPHA3, pFLAG‐CMV5a‐EPHA3ΔC, pFLAG‐CMV5a‐EPHA7, and pFLAG‐CMV5b‐EPHA7ΔC plasmids, the human *EPHA3* and *EPHA7* cDNAs were obtained by reverse transcription PCR using RNA extracted from LNCaP cells. Primers used and PCR cycling conditions are listed in Table S1. The PCR products were then cloned into the pFLAG‐CMV5a or pFLAG‐CMV5b vector (Sigma‐Aldrich, Saint Louis, MO, USA). All sequences inserted into the vector were confirmed by sequencing on an ABI Prism 3100xl Genetic Analyzer (Applied Biosystems, Waltham, MA, USA). Electroporation was used to introduce the plasmids into LNCaP cells with the Neon Transfection System (Invitrogen, Carlsbad, CA, USA) following the manufacturer's suggested protocol.

### 2.4 siRNA Transfection

siRNAs targeting stomatin, ephrin‐A5, EphA3, EphA7, ELK1, and ELK4, as well as a negative control RNA (Scramble), were produced using the CUGA7 *in vitro* transcription kit (Nippon Gene, Tokyo, Japan). The siRNA sequences were as follows:

Stomatin siRNA: 5′‐GGAGAUCCUCACAAAGGAU‐3′

Ephrin‐A5 siRNA: 5′‐CCUCUACAUGGUGAACUUU‐3′

EphA3 siRNA: 5′‐GCCUGACACUAUAUACGUA‐3′

EphA7 siRNA: 5′‐GCUAGAUGCCUCCUGUAUU‐3′

ELK1 siRNA: 5′‐CUGAAAUCGGAAGAGCUUAAU‐3′

ELK4 siRNA: 5′‐AGCCUAACAUGAAUUAUGACA‐3′

Scramble: 5′‐CAGUCGCGUUUGCGACUGG‐3′

The siRNA transfection procedures were performed using Lipofectamine RNAiMAX Transfection Reagent (Invitrogen), according to the manufacturer's instructions. Briefly, we performed the reverse transfection method in which LNCaP cells (4 × 10^5^ cells) or PC3M cells (4 × 10^5^ cells) were incubated with 40 nmol/L siRNA duplexes combined with Lipofectamine RNAiMAX Transfection Reagent.

### 2.5 Cell Proliferation Assay

LNCaP cells and PC3M cells were transfected as indicated. After 24 h since transfection, 2 × 10^4^ cells were plated on 35 mm dishes. Cells were counted using a cell counting chamber (Erma, Tokyo, Japan) at the indicated time points.

### 2.6 Antibodies

Antibodies used are listed in Table S2.

### 2.7 Co‐Culture of LNCaP Cells With PrS Cells

Co‐culture experiments were performed as described previously [11]. Briefly, EGFP‐labeled LNCaP cells (4 × 10^4^ cells) were co‐cultured with PrS cells (2 × 10^5^ cells) for 48 h or were cultured alone as a control. Cell culture‐conditioned media were mixed between dishes every 6 h. After the co‐culture, the EGFP‐labeled LNCaP cells were isolated from PrS cells using FACSAria flow cytometry (BD Biosciences, San Jose, CA, USA). The isolated LNCaP cells were lysed, and the extracts were used for qPCR and western blot analyses.

### 2.8 qPCR

Total RNA was extracted from cells using TRIzol reagent (Thermo Fisher Scientific, Waltham, MA, USA), according to the manufacturer's instructions. cDNA was synthesized using the ReverTra Ace qPCR RT Master Mix with gDNA Remover (Toyobo, Osaka, Japan). Then, qPCR experiments were performed using the LightCycler Instrument (Roche Diagnostics, Basel, Switzerland) as described previously [8]. The samples were run in duplicate, and the data were quantified by the standard curve method. The primers used are listed in Table S3.

### 2.9 Isolation of the Membrane Fraction

For the detection of endogenous stomatin expression, we isolated the membrane fraction of LNCaP cells and PC3M cells. Cells were washed in ice‐cold PBS and resuspended in subcellular fractionation buffer containing 20 mmol/L HEPES (pH 7.4), 10 mmol/L KCl, 2 mmol/L MgCl_2_, 200 mmol/L sucrose, 1 mmol/L ethylenediaminetetraacetic acid, 1 mmol/L ethyleneglycol tetraacetic acid, 2 mmol/L phenylmethylsulfonyl fluoride (PMSF), and 1 mg/L leupeptin [12]. The cells were then homogenized in the buffer with 10 strokes using the Potter‐Elvehjem tissue homogenizer (DWK Life Sciences, Millville, NJ, USA), followed by centrifugation at 700×*g* at 4°C for 5 min. The pellet was washed with the fractionation buffer, then passed through a 25‐gauge needle 10 times, followed by centrifugation at 700×*g* at 4°C for 10 min. These supernatants were transferred into a new tube and centrifuged at 10,000×*g* at 4°C for 5 min. The pellet was washed with the fractionation buffer, then passed through a 25‐gauge needle 10 times, followed by centrifugation at 10,000×*g* at 4°C for 10 min. These supernatants, including the membrane, were transferred into a new tube and centrifugated at 100,000×*g* at 4°C for 1 h. The pellet was resuspended in RIPA lysis buffer containing 1 mmol/L PMSF, 1 μg/mL leupeptin, 1 μg/mL aprotinin, 5 mmol/L sodium fluoride, and 1 mmol/L sodium orthovanadate to obtain the membrane fraction.

### 2.10 Immunoblot Analysis

Cells were lysed in RIPA lysis buffer containing 1 mmol/L PMSF, 1 μg/mL leupeptin, 1 μg/mL aprotinin, 5 mmol/L sodium fluoride, and 1 mmol/L sodium orthovanadate. The lysates were then centrifuged at 20,000×*g* for 10 min. The samples were separated by SDS‐PAGE, followed by blotting on polyvinylidene difluoride membranes (Bio‐Rad Laboratories, Hercules, CA, USA). The membrane was incubated overnight at 4°C with the indicated primary antibody. The antibodies used are listed in Table S2. The horseradish peroxidase (HRP)‐conjugated secondary antibody (GE Healthcare, Chicago, IL, USA) diluted in 5% skim milk was applied for 2 h at room temperature. To visualize the protein bands, the membrane was treated with Luminata Forte HRP substrate (Millipore, Billerica, MA, USA) for 5 min and observed on a luminescent image analyzer LAS‐4000 (Fujifilm Life Science, Tokyo, Japan). Band densities were analyzed using ImageJ software (National Institutes of Health, Bethesda, MD, USA).

### 2.11 Immunofluorescence Staining

For protein staining assays, cells grown on glass coverslips coated with fibronectin were fixed with 4% (w/v) paraformaldehyde for 15 min and permeabilized with 0.2% (w/v) Triton X‐100 for 5 min. Non‐specific staining was reduced by blocking with 3%–5% BSA for 30 min at room temperature. The samples were incubated overnight at 4°C with the indicated primary antibodies listed in Table S2. The fluorescently labeled secondary antibodies Alexa Fluor 488 and Alexa Fluor 555 (1:200–1000 dilution) (Thermo Fisher Scientific) were applied for 1 h at room temperature in the dark. After washing the samples with PBS, the nuclei were stained with DAPI (1:200 dilution) (Dojindo, Kumamoto, Japan) for 5 min. The cells were viewed using a Leica SP8 confocal microscope (Leica Microsystems, Wetzlar, Germany).

### 2.12 Analysis Using TCGA Database

*STOM* gene expression data and clinical follow‐up information of prostate cancer patients from The Cancer Genome Atlas (TCGA) database were downloaded via the University of California Santa Cruz (UCSC) Xena browser (https://xenabrowser.net/). A total of 539 prostate cancer patients were retrieved. Clinical follow‐up information included overall survival time, disease‐free time, and progression‐free time. The *STOM* RNA‐seq values were processed using the log2 (*x* + 1) conversion method. The low *STOM* expression group was defined as an *STOM* RNA‐seq value < 11.05 and the high *STOM* expression group was defined as a value ≥ 11.05. Kaplan–Meier analysis was performed after patients were divided into two groups based on the *STOM* expression level.

### 2.13 IHC Assays in Human Prostate Cancer Samples

Formalin‐fixed paraffin‐embedded prostate cancer samples were obtained from Shiga University of Medical Science Hospital with the approval of the Ethics Committee of Shiga University of Medical Science (No. R2017‐059). The samples were sectioned at 4 μm thickness, and deparaffinized using xylene. After deparaffinization, xylene is removed with 100% ethanol. The samples were hydrated and then soaked in pre‐heated antigen retrieval solution (100 mmol/L Tris–HCl [pH 8.3] and 10 mmol/L EDTA) at 80°C for 10 min. The samples were cooled down at room temperature, washed with PBS for 15 min, and permeabilized with 0.3% Triton X‐100 for 30 min. Non‐specific staining was reduced by blocking with 1% milk and 3% BSA for 30 min at room temperature. The samples were incubated overnight at 4°C in combination with the indicated primary antibodies. The antibodies used are listed in Table S2. The fluorescently labeled secondary antibodies Alexa Fluor 488 and Alexa Fluor 555 (1:800 dilution) (Thermo Fisher Scientific) were applied for 1 h at room temperature in the dark. After washing the samples with PBS, the nuclei were stained with DAPI (1:250 dilution) (Dojindo) for 5 min. The cells were viewed using a TCS SP8 X confocal microscope (Leica).

### 2.14 Mouse Xenograft Tumor Samples

LNCaP cells (1 × 10^7^ cells) in 200 μL of PBS containing Matrigel (50% v/v) (Corning Inc., Corning, NY, USA) were subcutaneously injected through a 25‐gauge needle into the dorsal surfaces of male NOD/ShiJic‐scid (NOD.CB17‐Prkdcscid/Jcl) mice aged 8–12 weeks. The mice were purchased from CLEA Japan, Inc. (Tokyo, Japan). After 10 weeks, the tumors were isolated from sacrificed mice. Cryosections of mouse xenograft tumors were fixed in 4% paraformaldehyde for 20 min, washed with PBS for 15 min, and stained with the indicated primary antibodies overnight at 4°C. The antibodies used are listed in Table S2. The fluorescently labeled secondary antibodies Alexa Fluor 488 and Alexa Fluor 555 (1:800 dilution) (Thermo Fisher Scientific) were applied for 1 h at room temperature in the dark. After washing the samples with PBS, the nuclei were stained with DAPI (1:250 dilution) (Dojindo) for 5 min. The cells were viewed using a TCS SP8 X confocal microscope (Leica). The animal experiments were approved by Shiga University of Medical Science Animal Care and Use Committee (No. 2021‐1‐6), and were performed in accordance with relevant guidelines and regulations including Animal Research Reporting of *In Vivo* Experiments (ARRIVE) guidelines.

### 2.15 Statistics

All data are expressed as the mean ± standard deviation. The experiments in each figure were performed at least three times, and statistical differences between experimental groups were evaluated using the two‐tailed Student's *t*‐test, one‐way analysis of variance (ANOVA) or two‐way repeated measures ANOVA. If ANOVA indicated overall significance, individual differences were evaluated using the Bonferroni's post‐test. Kaplan–Meier analysis was used to evaluate the prognosis of prostate cancer patients in two groups, and the obtained curves related to the prognosis were compared to the log‐rank test. *p* < 0.05 was considered a statistically significant difference.

## 3 Results

### 3.1 Suppression of Stomatin Expression by EphA–Ephrin‐A Signaling

Before exploring the regulatory mechanism of stomatin expression, we analyzed the clinical importance of the expression in the prognosis of prostate cancer using the TCGA database. The analysis showed that the disease‐free ratio and the progression‐free ratio were significantly better in the high stomatin expression group than the low stomatin expression group, although the overall survival ratio was similar between the groups (Figure S1). To examine the mechanism controlling the cell‐to‐cell contact‐dependent regulation of stomatin expression, we searched candidate genes expressed in LNCaP cells. We used two datasets in Harmonizome (https://maayanlab.cloud/Harmonizome/) [9], and selected the genes encoding cell surface proteins that can play a role in both intercellular communication and intracellular signal transduction. We found that *EPHA3*, *EPHA7*, and *EFNA5* were top‐ranked candidate genes (Table S4).

**FIGURE 1:**
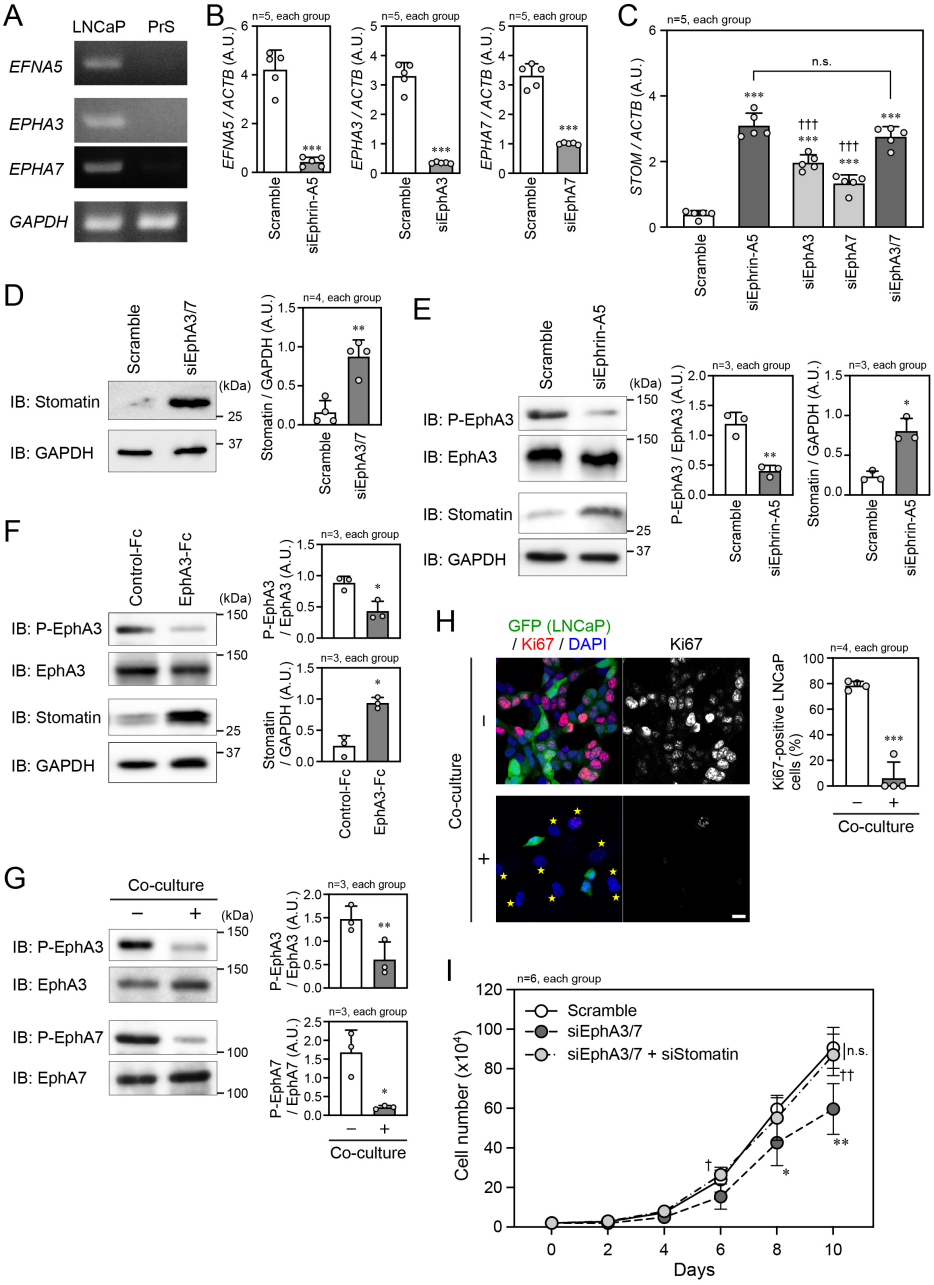
Suppression of stomatin expression by EphA–ephrin‐A signaling. (A) PCR analysis of *EFNA5*, *EPHA3*, and *EPHA7* gene expression levels in prostate cancer LNCaP cells and prostate stromal (PrS) cells. *GAPDH* was used as the internal control gene. (B) Confirmation of siRNA‐mediated knockdown of *EFNA5*, *EPHA3*, and *EPHA7* expressions in LNCaP cells by qPCR. The mRNA levels of the indicated genes were normalized to *ACTB* mRNA level. (C) qPCR to analyze *STOM* mRNA level in LNCaP cells after transfection of the indicated siRNAs. The *STOM* mRNA level was normalized to *ACTB* mRNA level. (D) (Left) Immunoblot (IB) analysis of stomatin protein expression in LNCaP cells transfected with scramble RNA or both siEphA3 and siEphA7 (siEphA3/7). (Right) Quantification of the relative stomatin band density in each group. The stomatin protein expression level was normalized to GAPDH protein expression level. (E) (Left) IB analysis of phosphorylated EphA3 (P‐EphA3) and stomatin expression in LNCaP cells transfected with scramble RNA or siEphrin‐A5. (Right) Quantification of the relative P‐EphA3 and stomatin band densities in each group. (F) (Left) IB analysis of P‐EphA3 and stomatin in LNCaP cells in the presence of control‐Fc or EphA3‐Fc (Right) Quantification of the relative P‐EphA3 and stomatin band densities in each group. (G) (Left) IB analysis of P‐EphA3 and P‐EphA7 in LNCaP cells co‐cultured with (+) or without (−) PrS cells for 48 h. (Right) Quantification of the relative P‐EphA3 and P‐EphA7 band densities in each group. (H) (Left) Confocal microscopic image of GFP‐labeled LNCaP cells co‐cultured with or without PrS cells. Nuclei were counterstained with DAPI. Stars indicate PrS cells. Scale bar, 20 μm. (Right) Quantification of Ki67‐positive LNCaP cells in each group. (I) The number of LNCaP cells at the indicated time points following transfection of scramble RNA, siEphA3/7, or both siEphA3/7 and siStomatin. In (B–I), *p < 0.05, **p < 0.01, and ***p < 0.001 vs. scramble RNA, control‐Fc, or co‐culture (−). ^††^ *p* < 0.01 and ^†††^ *p* < 0.001 vs. siEphA3/7. A. U., arbitrary unit. Statistical significance was determined by two‐tailed *t*‐test (B, D–H), one‐way ANOVA (C) and two‐way repeated measures ANOVA (I).

We next observed that EphA3 and EphA7 receptors, which are encoded by the *EPHA3* and *EPHA7* genes, respectively, are expressed in LNCaP cells, but not in PrS cells (Figure 1A). Additionally, their ligand ephrin‐A5, which is encoded by the *EFNA5* gene, showed the same cell‐specific expression pattern (Figure 1A). We next examined whether the stomatin expression level would be regulated by the EphA–ephrin‐A interaction between LNCaP cells. Following siRNA‐mediated knockdown of ephrin‐A5, EphA3, EphA7, or both EphAs in LNCaP cells, the *STOM* mRNA level was significantly elevated (Figure 1B,C). Notably, the magnitude of *STOM* mRNA elevation with ephrin‐A5 knockdown was similar to that with EphA3 and EphA7 (EphA3/7) simultaneous knockdown. However, knockdown of either EphA3 or EphA7 alone did not increase the *STOM* mRNA level to the same extent, suggesting the importance of both EphAs for stomatin expression regulation. We also confirmed that the EphA3/7 knockdown did not change the expression of other EphAs, and that similarly, the ephrin‐A5 knockdown did not affect the expression of other ephrin‐As in LNCaP cells (Figure S2). At the protein level, stomatin expression was also elevated by the simultaneous knockdown of EphA3/7 (Figure 1D). Similar to EphA3/7 knockdown, ephrin‐A5 knockdown increased the stomatin expression through the attenuation of EphA phosphorylation in LNCaP cells (Figure 1E). Further, we used EphA3‐Fc to inhibit the endogenous EphA–ephrin‐A interaction for its effect on stomatin expression. As expected, EphA3 phosphorylation was decreased and stomatin expression was increased in the presence of EphA3‐Fc (Figure 1F). When LNCaP cells were co‐cultured with PrS cells, which lost cell‐to‐cell contact between neighboring LNCaP cells and disrupted the EphA–ephrin‐A interaction, the phosphorylation of EphA3 and EphA7 was certainly suppressed (Figure 1G), and the co‐culture was shown to increase stomatin expression in our previous study [8]. In addition, the co‐culture significantly suppressed cell proliferation detected by the Ki67 signal (Figure 1H), which is consistent with the results that the co‐culture raised the expression of tumor‐suppressive molecule stomatin. Following these experiments, we examined the effect of EphA‐mediated signaling on cell proliferation. When EphA3/7 were knocked down together in LNCaP cells, the cell proliferation rate decreased. This lower rate was rescued by the additional knockdown of stomatin (Figure 1I). Taken together, these findings indicate that the attenuation of EphA signaling increases stomatin expression, which suppresses LNCaP cell proliferation.

### 3.2 The Effect of EphA‐Mediated Intracellular Signaling on Stomatin Expression

**FIGURE 2:**
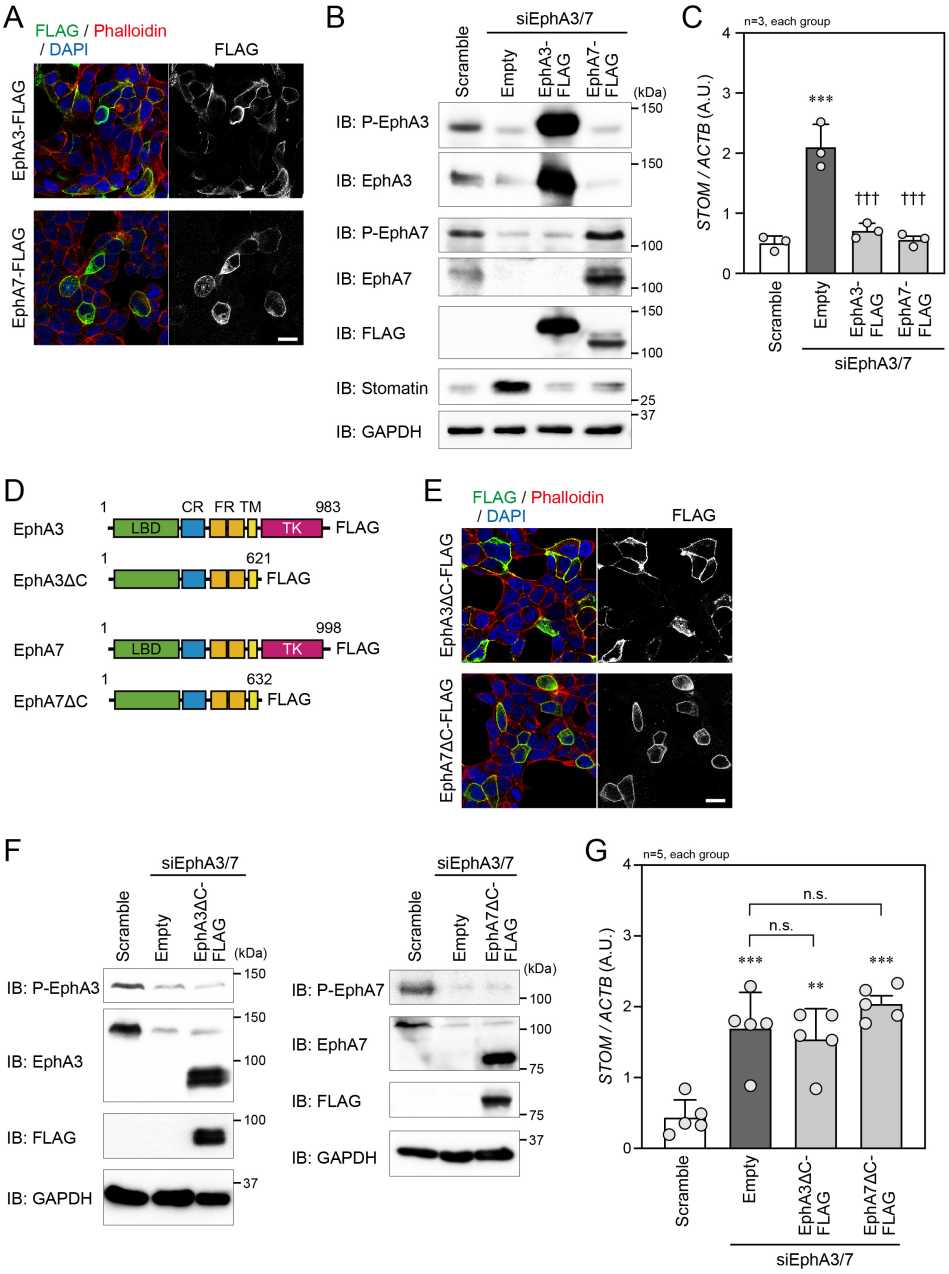
The effect of EphA‐mediated intracellular signaling on stomatin expression. (A) Confocal microscopic image of LNCaP cells transfected with the EphA3‐FLAG or EphA7‐FLAG. Scale bar, 20 μm. (B) IB analysis of LNCaP cell lysates following transfection of the indicated siRNAs and plasmids. GAPDH was used as the loading control. (C) qPCR to analyze *STOM* mRNA level in LNCaP cells described in (B). *STOM* mRNA level was normalized to *ACTB* mRNA level. (D) Schematic models of EphA3, EphA3ΔC, EphA7 and EphA7ΔC fused to the FLAG tag. The numbers indicate the amino acid positions. The green, blue, orange, yellow, and pink boxes show the ligand binding domain (LBD), cysteine‐rich domain (CR), fibronectin type III repeats (FR), transmembrane region (TM), and tyrosine kinase domain (TK), respectively. (E) Confocal microscopic image of LNCaP cells transfected with the EphA3ΔC‐FLAG or EphA7ΔC‐FLAG expression plasmid. Scale bar, 20 μm. (F) IB analysis of LNCaP cell lysates following transfection of the indicated siRNAs and plasmids. (G) qPCR to analyze *STOM* mRNA level in LNCaP cells described in (F). *STOM* mRNA level was normalized to *ACTB* mRNA level. In (C and G), **p < 0.01 and ***p < 0.001 vs. scramble RNA. ^†††^ *p* < 0.001 vs. empty plasmid. A. U., arbitrary unit. Statistical significance was determined by one‐way ANOVA.

Because ephrin‐A does not have the cytoplasmic domain, we focused on EphA to explore the intracellular signaling mechanism that regulates stomatin expression by the EphA–ephrin‐A interaction. For this purpose, we constructed plasmids expressing the C‐terminally FLAG‐tagged full‐length EphA3 (EphA3‐FLAG) or EphA7 (EphA7‐FLAG). When the plasmids were transfected into LNCaP cells, EphA3‐FLAG, and EphA7‐FLAG were confirmed to be localized on the plasma membrane (Figure 2A). We then performed rescue experiments using these plasmids in EphA3/7‐knockdown LNCaP cells. In the transfected cells, EphA3‐FLAG or EphA7‐FLAG was abundantly expressed, compared with endogenous EphA3 or EphA7, respectively (Figure 2B). The phosphorylation of EphA3 or EphA7 was also highly detected (Figure 2B). In these rescue experiments, the increase in stomatin expression induced by knocking down both EphA3 and EphA7 was almost completely suppressed by exogenously introducing EphA3‐FLAG or EphA7‐FLAG (Figure 2B,C). This suggests that the highly expressed EphA3‐FLAG or EphA7‐FLAG molecules could sufficiently participate in the endogenous signaling mechanism to suppress stomatin expression. Next, to examine whether the tyrosine kinase domain of EphA, which plays a role in the intracellular signaling, mediates the suppression of stomatin expression, we generated FLAG‐tagged EphA3 and EphA7 mutants lacking this domain (EphA3ΔC‐FLAG and EphA7ΔC‐FLAG) (Figure 2D). When expressed in LNCaP cells, EphA3ΔC‐FLAG and EphA7ΔC‐FLAG were localized on the plasma membrane (Figure 2E). Similar to EphA3‐FLAG or EphA7‐FLAG transfection in EphA3/7‐knockdown LNCaP cells, abundant expression of transfected EphA3ΔC‐FLAG or EphA7ΔC‐FLAG was detected in these cells (Figure 2F). However, EphA3ΔC‐FLAG or EphA7ΔC‐FLAG did not attenuate the stomatin expression increase induced by the simultaneous knockdown of EphA3/7 (Figure 2G). These results suggest that the tyrosine kinase domain of EphA3 or EphA7 is necessary for the signal transduction to suppress stomatin expression.

### 3.3 Regulation of Stomatin Expression by ERK Downstream of EphA

**FIGURE 3:**
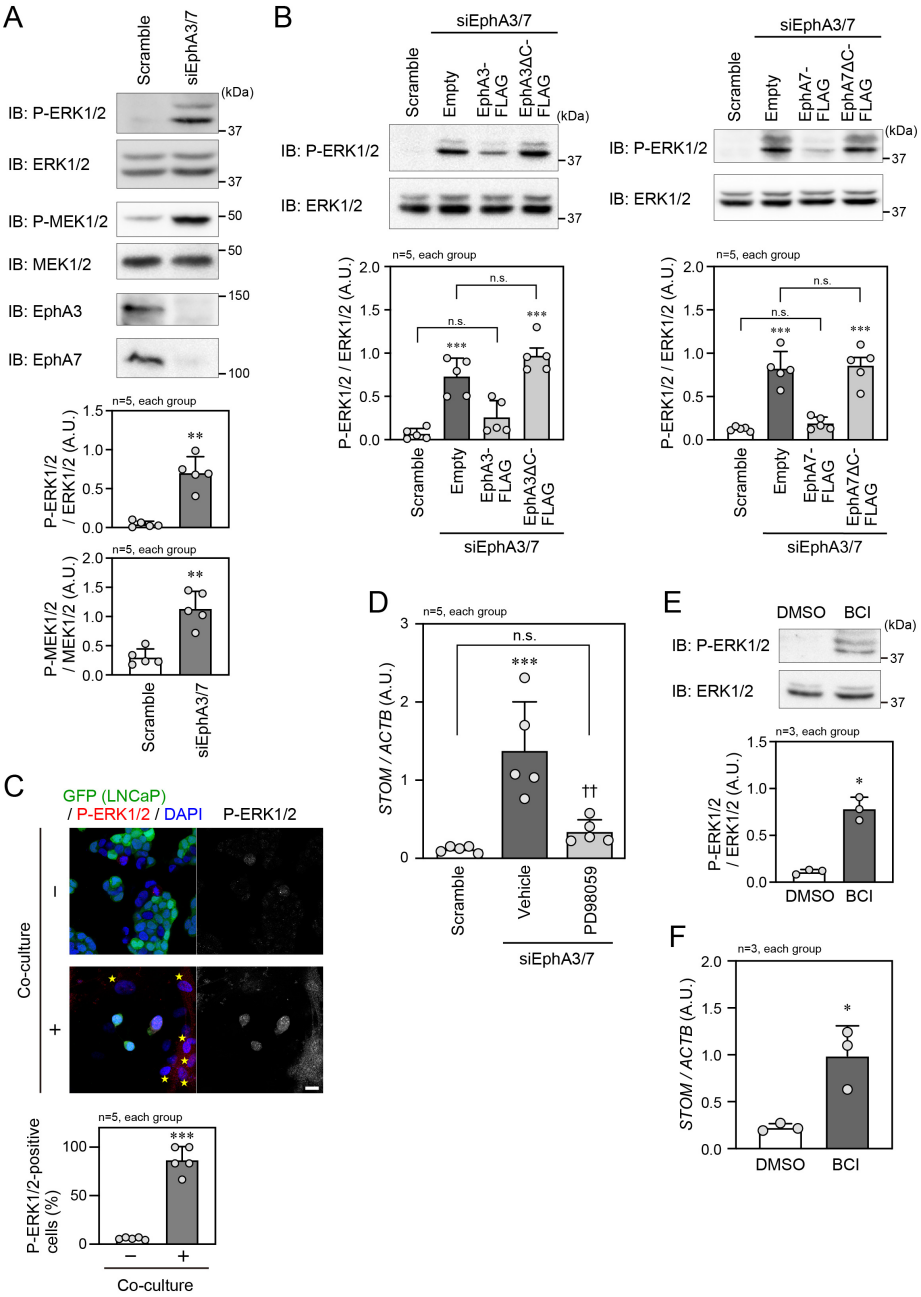
Regulation of stomatin expression by ERK. (A) (Top) IB analysis of LNCaP cell lysates following transfection with scramble RNA or siEphA3/7. (Bottom) Quantification of the relative phosphorylated ERK1/2 (P‐ERK1/2) and MEK1/2 (P‐MEK1/2) band densities in each group. (B) (Top) IB analysis of P‐ERK1/2 in LNCaP cell lysates following transfection of the indicated siRNAs and plasmids. (Bottom) Quantification of the relative P‐ERK1/2 band density in each group. (C) (Left) Confocal microscopic image of GFP‐labeled LNCaP cells co‐cultured with or without PrS cells. Nuclei were counterstained with DAPI. Stars indicate PrS cells. Scale bar, 20 μm. (Right) Quantification of P‐ERK‐positive LNCaP cells in each group. (D) qPCR analysis of *STOM* mRNA expression level in LNCaP cells transfected with scramble RNA or siEphA3/7 and treated with or without PD98059. *STOM* mRNA level was normalized to *ACTB* mRNA level. (E) (Top) IB analysis of LNCaP cell lysates following treatment with control DMSO or BCI. (Bottom) Quantification of the relative P‐ERK1/2 band density in each group. (F) qPCR analysis of *STOM* mRNA expression level in LNCaP cells treated with control DMSO or BCI. *p < 0.05, **p < 0.01, and ***p < 0.001 vs. scramble RNA, co‐culture (−), or DMSO. ^††^ *p* < 0.01 vs. vehicle. A. U., arbitrary unit. Statistical significance was determined by two‐tailed *t*‐test (A, C, E, and F) and one‐way ANOVA (B and D).

The ephrin‐mediated activation of Eph receptors reportedly leads to inactivation of the Ras/MEK/ERK pathway in fibroblasts, epithelial cells, neuronal cells, and myoblasts as well as in cancer cells [13, 14, 15, 16, 17, 18]. Therefore, we examined whether EphA3/7‐mediated inactivation of the ERK signaling pathway in LNCaP cells could result in suppression of stomatin expression. Knockdown of both EphA3 and EphA7induced phosphorylation of ERK1/2 and MEK1/2 in LNCaP cells (Figure 3A). The ERK1/2 phosphorylation was attenuated by the additional transfection of EphA3‐FLAG or EphA7‐FLAG, but not by transfection of EphA3ΔC‐FLAG or EphA7ΔC‐FLAG (Figure 3B). In contrast, the ERK1/2 phosphorylation was enhanced by co‐culture of LNCaP cells with PrS cells (Figure 3C). The increased stomatin expression observed following EphA3/7 knockdown did not occur when the cells were treated with a MAPK/ERK kinase inhibitor, PD98059 (Figure 3D). Dual specificity phosphatase 6 (DUSP6) has been previously shown to antagonize ERK activity, while a DUSP6 small molecule inhibitor, (E/Z)‐BCI hydrochloride (BCI), induces ERK activation [19, 20, 21]. Therefore, we tested the effects of BCI‐induced ERK activation on stomatin expression. Treating the cells with BCI certainly induced the activation of ERK1/2 (Figure 3E) and promoted stomatin expression (Figure 3F). These findings suggest that the ERK pathway, which is attenuated by the EphA‐mediated signaling, positively regulates stomatin expression in LNCaP cells.

### 3.4 Effect of the ERK–ELK Signaling Pathway on Stomatin Expression

**FIGURE 4:**
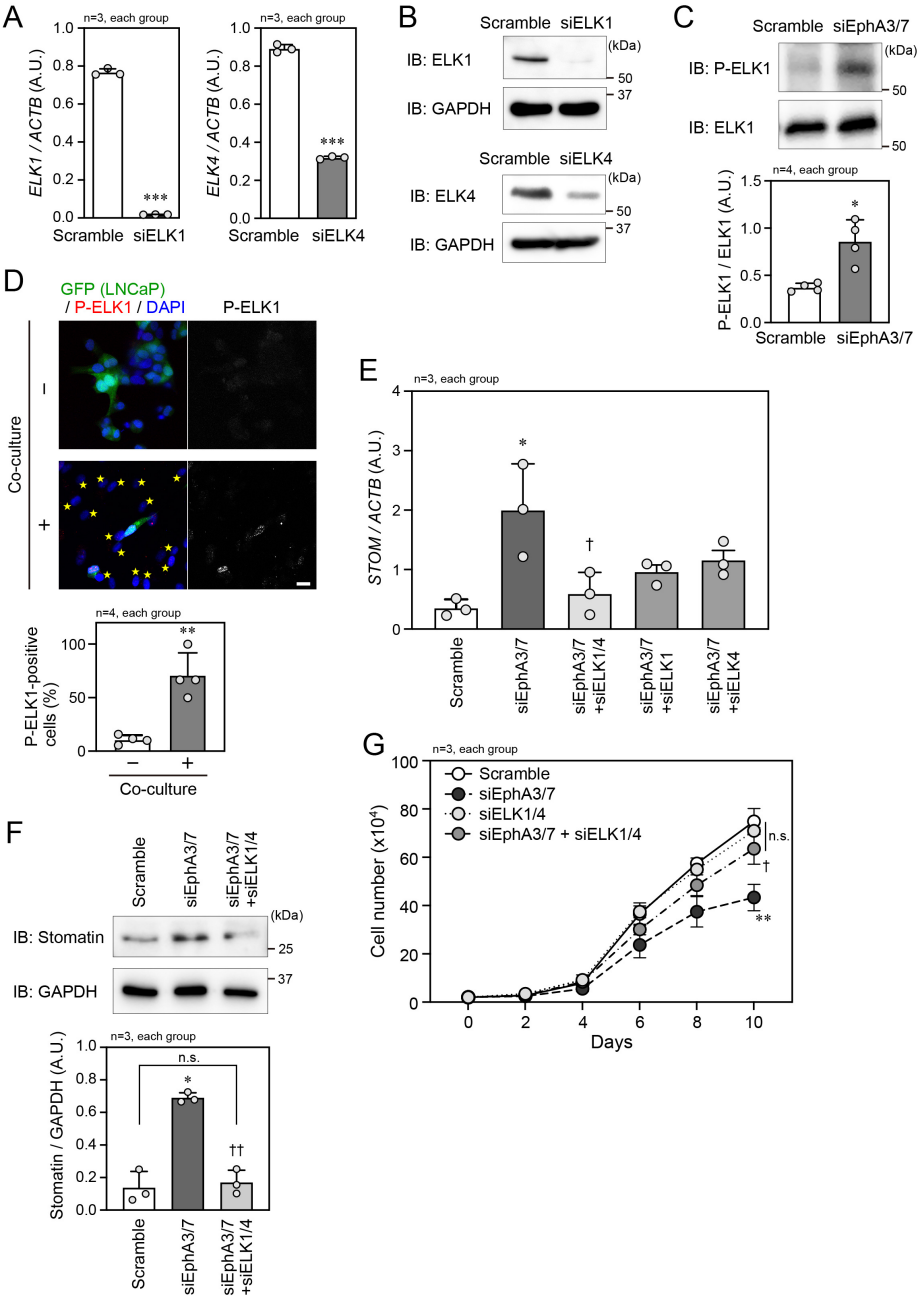
Effect of ERK–ELK signaling on stomatin expression. (A) Confirmation of siRNA‐mediated knockdown of *ELK1* and *ELK4* expression in LNCaP cells by qPCR. (B) IB analysis of LNCaP cell lysates following transfection with scramble RNA, siELK1, or siELK4. GAPDH was used as the loading control. (C) (Top) IB analysis of phosphorylated ELK1 (P‐ELK1) and total ELK1 expression in LNCaP cells transfected with scramble RNA or siEphA3/7. (Bottom) Quantification of the relative P‐ELK1 band density in each group. (D) (Top) Confocal microscopic image of GFP‐labeled LNCaP cells co‐cultured with or without PrS cells. Nuclei were counterstained with DAPI. Stars indicate PrS cells. Scale bar, 20 μm. (Bottom) Quantification of P‐ELK1‐positive LNCaP cells in each group. (E) qPCR analysis of *STOM* mRNA level in LNCaP cells transfected with scramble RNA, siEphA3/7, or in combination with siEphA3/7 and siELK1/4, siELK1 or siELK4. *STOM* mRNA level was normalized to *ACTB* mRNA level. (F) (Top) IB analysis of stomatin protein expression in LNCaP cells transfected with scramble RNA, siEphA3/7, or both siEphA3/7 and siELK1/4. (Bottom) Quantification of the relative stomatin band density in each group. (G) The number of LNCaP cells at the indicated time points after transfection with scramble RNA, siEphA3/7, siELK1/4, or both siEphA3/7 and siELK1/4. In (A and C–G), *p < 0.05, **p < 0.01, and ***p < 0.001 vs. scramble RNA or co‐culture (−). ^†^ *p* < 0.05 and ^††^ *p* < 0.01 vs. siEphA3/7. A. U., arbitrary unit. Statistical significance was determined by two‐tailed *t*‐test (A, C, and D), one‐way ANOVA (E and F), and two‐way repeated measures ANOVA (G).

ERK is known to directly phosphorylate hundreds of substrates that are localized in either the cytoplasm, various organelles, or nucleus [22, 23]. We used the PROMO website (https://alggen.lsi.upc.es/cgi‐bin/promo_v3/promo/promoinit.cgi?dirDB=TF_8.3) to search for ERK substrates that can regulate stomatin expression. Several predicted binding sites of the ELK family transcription factors that function downstream of ERK are present in a 5 kb sequence of the *STOM* promoter region (Figure S3). Because LNCaP cells endogenously express ELK1 and ELK4, siRNAs efficiently targeting ELK1 and ELK4 were then generated (Figure 4A,B). Simultaneous knockdown of EphA3/7 induced phosphorylation of ELK1 (Figure 4C), suggesting that the ERK–ELK pathway is suppressed by EphA‐mediated signaling in LNCaP cells. In line with this, the ELK1 phosphorylation was increased when LNCaP cells were co‐cultured with PrS cells to inhibit the EphA–ephrin‐A interaction‐induced EphA‐mediated signaling (Figure 4D). Moreover, the increase in stomatin expression following EphA3/7 knockdown was abrogated at both the mRNA and protein levels by additional knockdown of both ELK1 and ELK4 (ELK1/4) (Figure 4E,F). Either ELK1 or ELK4 knockdown in addition to EphA3/7 knockdown was not sufficient to fully suppress stomatin expression (Figure 4E). The reduced cell proliferation rate following EphA3/7 knockdown via increased stomatin expression was restored by additional knockdown of ELK1/4, although ELK1/4 knockdown itself did not affect the cell proliferation rate (Figure 4G). Taken together, we conclude that the EphA–ephrin‐A system negatively regulates stomatin expression via suppression of the ERK–ELK signaling axis in LNCaP cells.

### 3.5 EphA‐Mediated Modulation of Stomatin Expression in Another Prostate Cancer Cell Type

**FIGURE 5:**
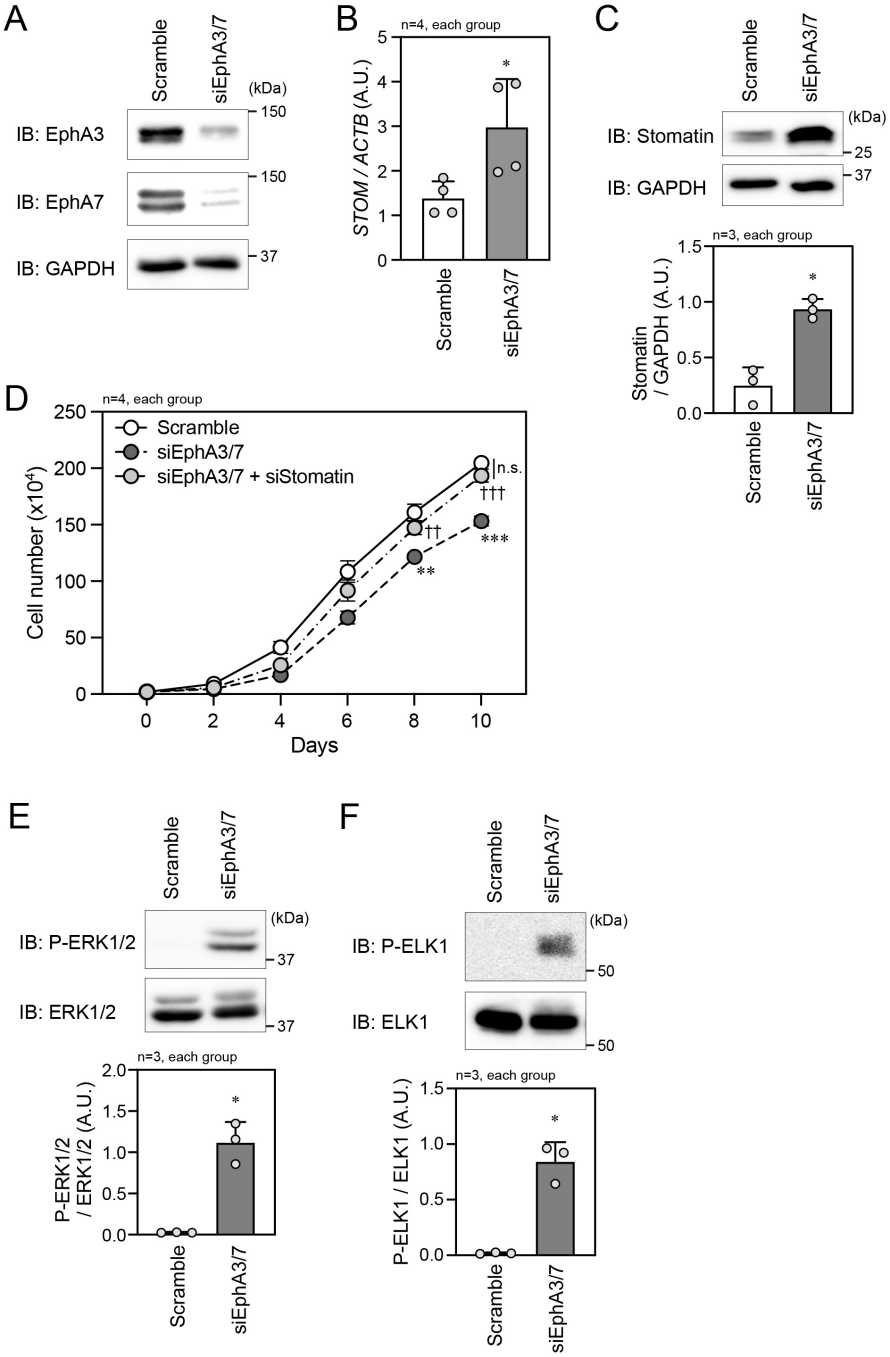
EphA‐mediated regulation of stomatin expression in PC3M cells. (A) IB analysis of EphA3 and EphA7 expression and confirmation of siRNA‐mediated knockdown of the expression in PC3M cells. GAPDH was used as the loading control. (B) qPCR analysis of *STOM* mRNA level in PC3M cells transfected with scramble RNA or siEphA3/7. *STOM* mRNA level was normalized to *ACTB* mRNA level. (C) (Top) IB analysis of stomatin protein expression in PC3M cells transfected with scramble RNA or siEphA3/7. (bottom) Quantification of the relative stomatin band density in each group. (D) The number of PC3M cells at the indicated time points after transfection with scramble RNA, siEphA3/7, or both siEphA3/7 and siStomatin. (E and F) (Top) IB analysis of P‐ERK1/2 (E) and P‐ELK1 (F) in PC3M cells transfected with scramble RNA or siEphA3/7. (Bottom) Quantification of the relative P‐ERK1/2 (E) and P‐ELK1 (F) band densities in each group. In (B–F), *p < 0.05, **p < 0.01, and ***p < 0.001 vs. scramble RNA. ^††^ *p* < 0.01 and ^†††^ *p* < 0.001 vs. siEphA3/7. A. U., arbitrary unit. Statistical significance was determined by two‐tailed *t*‐test (B, C, E, and F) and two‐way repeated measures ANOVA (D).

To increase the generality of the regulatory model for stomatin expression, we additionally used another prostate cancer cell type PC3M cells in the experiments. First, we confirmed the EphA3 and EphA7 expressions in PC3M cells and the knockdown efficiency of siEphA3/7 in the cells (Figure 5A). Similar to LNCaP cells, the stomatin expression was significantly increased by knockdown of EphA3/7 in PC3M cells (Figure 5B,C). The cell proliferation rate of PC3M cells was also suppressed by EphA3/7 knockdown, and the rate was restored by additional knockdown of stomatin (Figure 5D). In the intracellular signaling system, EphA3/7 knockdown certainly increased the phosphorylation of ERK1/2 and ELK1 in PC3M cells (Figure 5E,F). All of these results are similarly observed in LNCaP cells, and thus, the molecular mechanism on EphA‐mediated modulation of stomatin expression seems to be common in prostate cancer cells.

### 3.6 Association of Stomatin Expression With EphA3, ERK, and ELK Phosphorylation in Human Prostate Cancer

**FIGURE 6:**
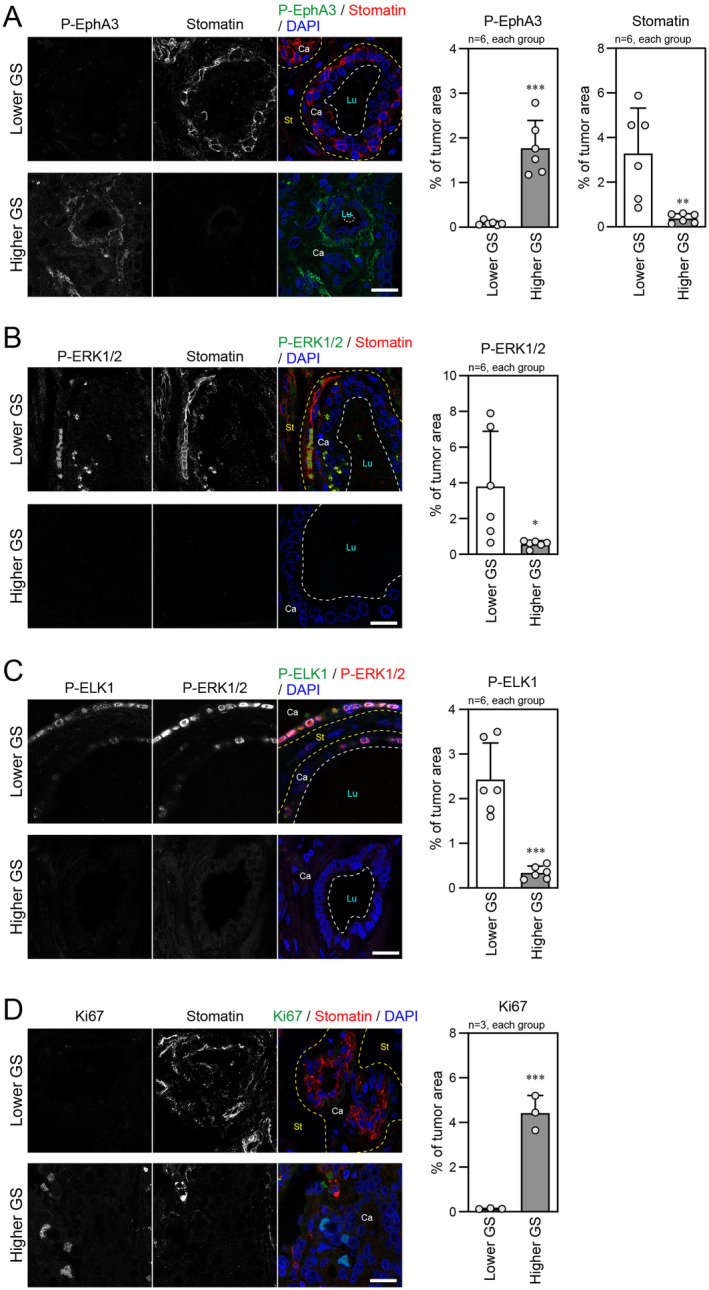
IHC analysis of stomatin, Ki67, and phosphorylated EphA3, ERK1/2, and ELK1 in human prostate cancer samples. (A–D) (Left panels) Representative IHC images of human prostate cancer samples from patients with lower or higher Gleason score (GS). Co‐staining of P‐EphA3 and stomatin (A), P‐ERK1/2 and stomatin (B), P‐ELK1 and P‐ERK1/2 (C), and Ki67 and stomatin (D). The nuclei were counterstained with DAPI. Scale bars, 20 μm. Yellow dotted lines indicate the border between cancer and stoma areas, and white dotted lines indicate lumens. Ca, cancer area; St, stromal area; Lu, lumen (right panels) Percentage of each IHC staining signal in the tumor area. *p < 0.05, **p < 0.01, and ***p < 0.001 vs. lower GS, which was determined by two‐tailed *t*‐test.

To extend the above *in vitro* findings to clinical implications, we performed IHC assays in human prostate cancer samples from patients with lower or higher GS. EphA3 phosphorylation was often observed in higher GS samples compared with in lower GS samples (Figure 6A). This was consistent with a previous publication that showed that EphA3 contributes to malignant progression of human prostate cancer with its increased expression level observed in higher GS samples [24]. The opposite trend was seen for stomatin. The increased stomatin expression was found in the lower GS samples in which the stroma was in contact with the tumor region, but the decreased expression in the higher GS samples filled with the tumor cells alone (Figure 6A,B,D). These data corroborated the results of our *in vitro* experiments in this study and those from our previous report [8]. The tumor cells expressing phosphorylated ERK1/2 in the lower GS samples coincided with those expressing stomatin, while few tumor cells expressing both phosphorylated ERK1/2 and stomatin were observed in the higher GS samples (Figure 6B). We also found increased level of phosphorylated ELK1, which was often co‐localized with phosphorylated ERK1/2 in the tumor cell nuclei, in the lower GS samples, compared with the higher GS samples (Figure 6C). Moreover, in lower GS samples with high stomatin expression, tumor cells positive for the proliferation marker Ki67 were hardly detected, while in higher GS samples, Ki67‐positive cells were often observed (Figure 6D). These findings suggest that the regulation of stomatin expression by the EphA‐modulated ERK–ELK signaling axis may be associated with the malignancy of human prostate cancer.

### 3.7 Verification of the EphA‐Modulated ERK–ELK Signaling Axis and Stomatin Expression in the Mouse Xenograft Tumor Samples

**FIGURE 7:**
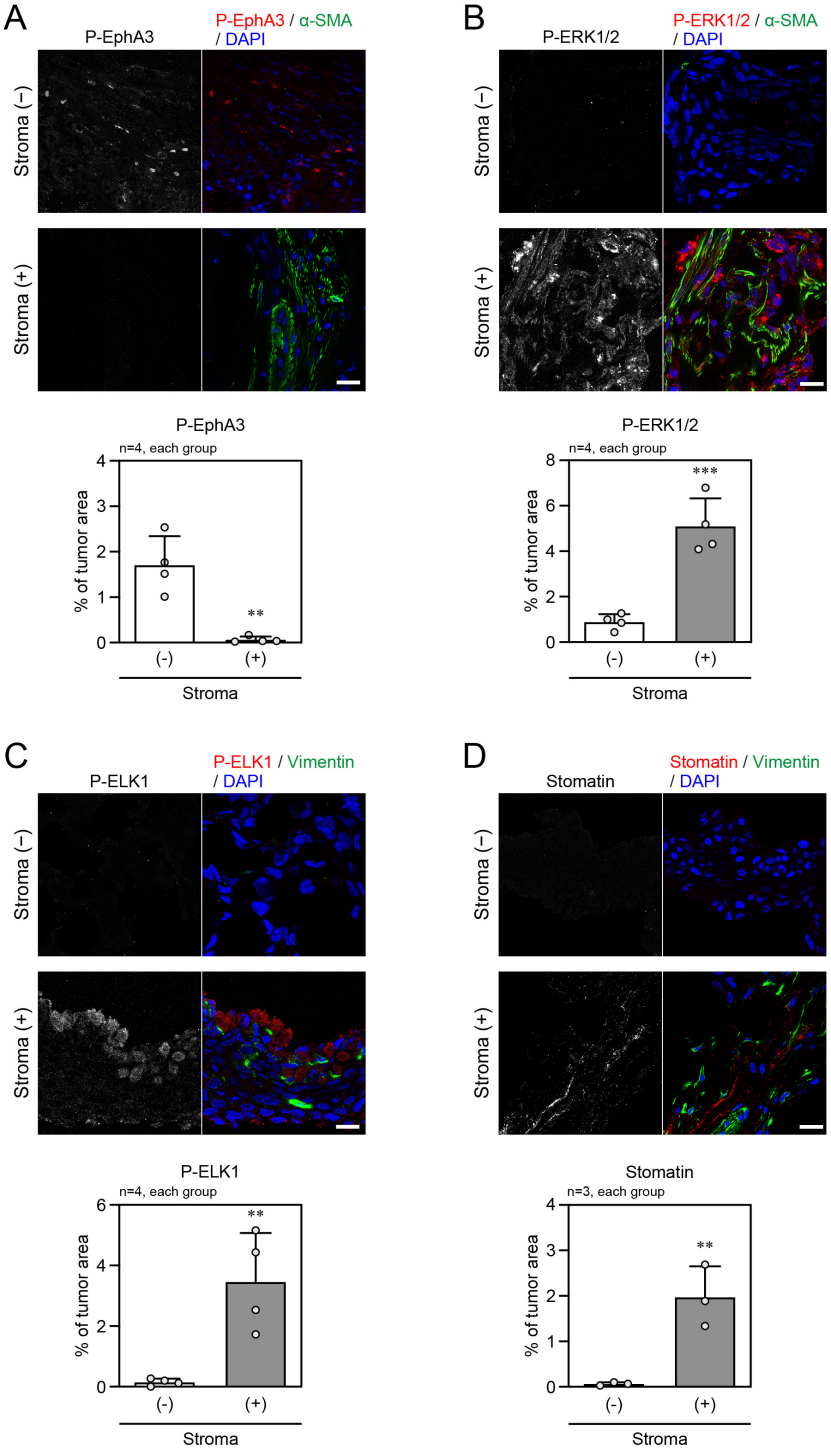
IHC analysis in mouse xenograft tumor samples. (A–D) (Top panels) Representative IHC images of xenograft tumor samples from NOD/SCID mice in cancer areas apart from stromal areas [Stroma (−)] or close to stromal areas [Stroma (+)]. Co‐staining of P‐EphA3 and a stromal cell marker α‐smooth muscle actin (α‐SMA) (A), P‐ERK1/2 and α‐SMA (B), P‐ELK1 and another stromal cell marker vimentin (C), and stomatin and vimentin (D). The nuclei were counterstained with DAPI. Scale bars, 20 μm. (Bottom panels) Percentage of each IHC staining signal in the tumor area. **p < 0.01, and ***p < 0.001 vs. Stroma (−), which was determined by two‐tailed *t*‐test.

Finally, IHC assays were performed using the xenograft tumor samples generated by implantation of LNCaP cells into NOD/SCID mice. EphA3 phosphorylation highly detected in the cancer cells apart from the stroma, which was stained with α‐SMA or vimentin, was attenuated in the area where stromal cells infiltrated the tumor (Figure 7A). In contrast, the phosphorylated ERK1/2, phosphorylated ELK1 and stomatin expression were low in the area of cancer cells alone, but were significantly high in cancer cells close to stromal cells (Figure 7B–D). These results confirmed our findings that the EphA signaling modulated the ERK–ELK signaling axis and stomatin expression, which was observed in our *in vitro* experiments.

## 4 Discussion

In this study, we elucidated the molecular mechanism of the cell‐to‐cell contact‐dependent regulation of *STOM* gene expression in prostate cancer cells. In these cells, ephrin‐A binding to EphA‐induced intracellular signal transduction that suppressed stomatin expression. When the EphA–ephrin‐A binding between the prostate cancer cells was interrupted by interactions with other cell types, such as PrS cells, or by knockdown of EphA3/7 or ephrin‐A5, ERK1/2 was activated to increase stomatin expression. Activated ERK1/2 phosphorylated ELK1 and ELK4 to promote transcriptional activation of the *STOM* gene. This regulatory mechanism was confirmed in both *in vitro* experiments and *in vivo* mouse xenograft tumor model. In prostate cancer samples from patients with lower GS, stomatin expression was enhanced, along with decreased EphA3 phosphorylation and increased ERK1/2 and ELK1 phosphorylation. The opposite results were observed in higher GS samples. These findings suggest the clinical significance of EphA3, ERK1/2 and ELK1 phosphorylation, and stomatin expression patterns in prostate cancer progression and malignancy.

Eph receptor tyrosine kinases and their ligands ephrins play a role in the regulation of tumor progression [25]. However, how Eph is involved in this regulation remains controversial. Eph inhibits the expansion and invasiveness of tumors, indicating the tumor suppressive effect of Eph‐induced signaling [26, 27]. In contrast, other reports have shown that activated Eph signaling supports cancer progression [17, 24, 28]. In this study, we found that the prostate cancer cells expressed both Eph ligand (ephrin‐A5) and receptors (EphA3/7), and that EphA‐mediated signaling in the cells inhibited stomatin expression, facilitating cancer cell proliferation. Conversely, simultaneous knockdown of EphA3/7induced stomatin expression via ERK1/2 and ELK1 phosphorylation, which inhibited cancer cell proliferation. Therefore, in the prostate cancer cells, mechanisms involving EphA3 and EphA7 contribute to tumor progression. These *in vitro* findings were supported by the data showing that EphA3 phosphorylation was increased in higher GS human prostate cancer samples. In malignant tumor, stromal cells often infiltrate the tumor, and the tumor cell phenotype is affected by these stromal cells. The expression of molecules including stomatin in cancer cells is regulated by this context. As shown in Figure 6, the stomatin expression was increased in lower GS prostate cancer samples in which stromal cells obviously infiltrated the tumor, while the expression was decreased in higher GS samples with less or without stromal cell infiltration.

It may be intriguing that expression of stomatin, a tumor suppressive molecule, was induced by a tumor proliferative signaling pathway like the MAP kinase cascade. It has recently been reported that the tumor suppressive molecules ARF and INK4a (ARF/INK4a), which are both encoded on the cyclin‐dependent kinase inhibitor 2A (*CDKN2a*) locus, are transcriptionally up‐regulated by proliferative or oncogenic signals, including RAS, Myc, and E2F [29]. This phenomenon has been called oncogene‐induced senescence which is a strong anti‐proliferative response induced by oncogenic signaling [30, 31]. Similarly, the transmembrane protein KIRREL1 functions as a tumor suppressor and is a direct target of Yes‐associated protein (YAP) and WW domain‐containing transcriptional regulator 1 (WWTR1; also known as TAZ) in the pro‐tumor Hippo signaling pathway [32]. Consistent with ARF/INK4a and KIRREL1, stomatin exerts a tumor suppressive effect by inhibiting PDPK1–Akt signaling. Therefore, our data reasonably suggest that stomatin expression is controlled by the mitogenic MAP kinase cascade as part of negative feedback loop in proliferative prostate cancer cells that are in active contact with surrounding stromal cells.

Scaffold and integral membrane proteins, including stomatin, were originally characterized as structural components of the plasma membrane that maintain cell morphology. These proteins have been also shown to act as regulators of intracellular signal transduction in cancer [33, 34, 35]. Members of the protein 4.1 family are membrane‐tethering proteins that localize just beneath the inner plasma membrane, and physically connect cell surface molecules to the actin cytoskeleton. Some of the members are also known to serve as tumor suppressors like stomatin by regulating intracellular signaling. Among them, protein 4.1N binds to protein phosphatase 1 and enhances its phosphatase activity to negatively regulate c‐Jun N‐terminal kinase (JNK). This results in inhibited growth of metastatic non‐small cell lung cancer cells [36]. In addition, protein 4.1B interacts with protein arginine N‐methyltransferase 3 (PRMT3) and down‐regulates PRMT3‐mediated post‐translational methylation, acting as a potentially important mechanism by which protein 4.1B suppresses tumor growth [37]. These findings provide an interesting notion that scaffold and integral membrane proteins may become attractive targets for anti‐cancer therapy, in addition to well‐studied targets of cytoplasmic signaling molecules and membrane receptors. Overall, stomatin is an emerging molecule that has both membrane‐tethering and tumor suppressive properties. In this study, we uncovered the specific molecular mechanism controlling its expression regulation. Our data will be helpful and insightful for the development of a potential novel anti‐cancer therapeutic approach that enhances the expression of the tumor suppressor stomatin in cancer cells.

## Author Contributions

**Masanari Nishida:** conceptualization (equal), data curation (equal), formal analysis (lead), investigation (lead), methodology (equal), writing – original draft (equal). **Akira Sato:** conceptualization (equal), data curation (equal), formal analysis (equal), investigation (equal), methodology (equal), writing – original draft (equal). **Akio Shimizu:** data curation (supporting), formal analysis (supporting), investigation (supporting), writing – review and editing (equal). **Nor Idayu A. Rahman:** data curation (equal), formal analysis (equal), investigation (equal), methodology (equal). **Akinori Wada:** methodology (supporting), writing – review and editing (supporting). **Susumu Kageyama:** methodology (supporting), writing – review and editing (supporting). **Hisakazu Ogita:** conceptualization (equal), data curation (equal), formal analysis (equal), funding acquisition (lead), investigation (equal), methodology (equal), supervision (lead), writing – original draft (equal), writing – review and editing (equal).

## Ethics Statement

Approval of the research protocol by an institutional review board: This study was conducted according to the guidelines of the Declaration of Helsinki and approved by the Ethics Committee of Shiga University of Medical Science (No. R2017‐059).

## Consent

The informed consent was obtained from the subjects whose samples were used for analysis after the operation in the form of opt‐out on the website of Shiga University of Medical Science Hospital.

## Conflicts of Interest

The authors declare no conflicts of interest.

## Animal Studies

The animal experiments conducted in this study were approved by Shiga University of Medical Science Animal Care and Use Committee (No. 2021‐1‐6), and were performed in accordance with relevant guidelines and regulations including Animal Research Reporting of *In Vivo* Experiments (ARRIVE) guidelines.

## Supporting information


**Figure S1.** Prognosis of prostate cancer patients with high or low stomatin expression.

**Figure S2.** The qPCR analysis for the expression of several *EPHA* and *EFNA* genes.

**Figure S3.** The sequence of the *STOM* gene promoter region.

**Table S1.** The primer sequences and PCR conditions for gene amplification.

**Table S2.** The primary antibodies used in this study.

**Table S3.** The primers for qPCR analysis.

**Table S4.** Gene expression profile in LNCaP cells.

## Data Availability

The data that support the findings of this study are available from the corresponding author upon reasonable request.
