# Supplementary figures and images for: EphA‐Mediated Regulation of Stomatin Expression in Prostate Cancer Cells
Source: Cancer Med. 2024 Oct 8;13(19):e70276. doi: 10.1002/cam4.70276 (PMC11459579; doi:10.1002/cam4.70276)

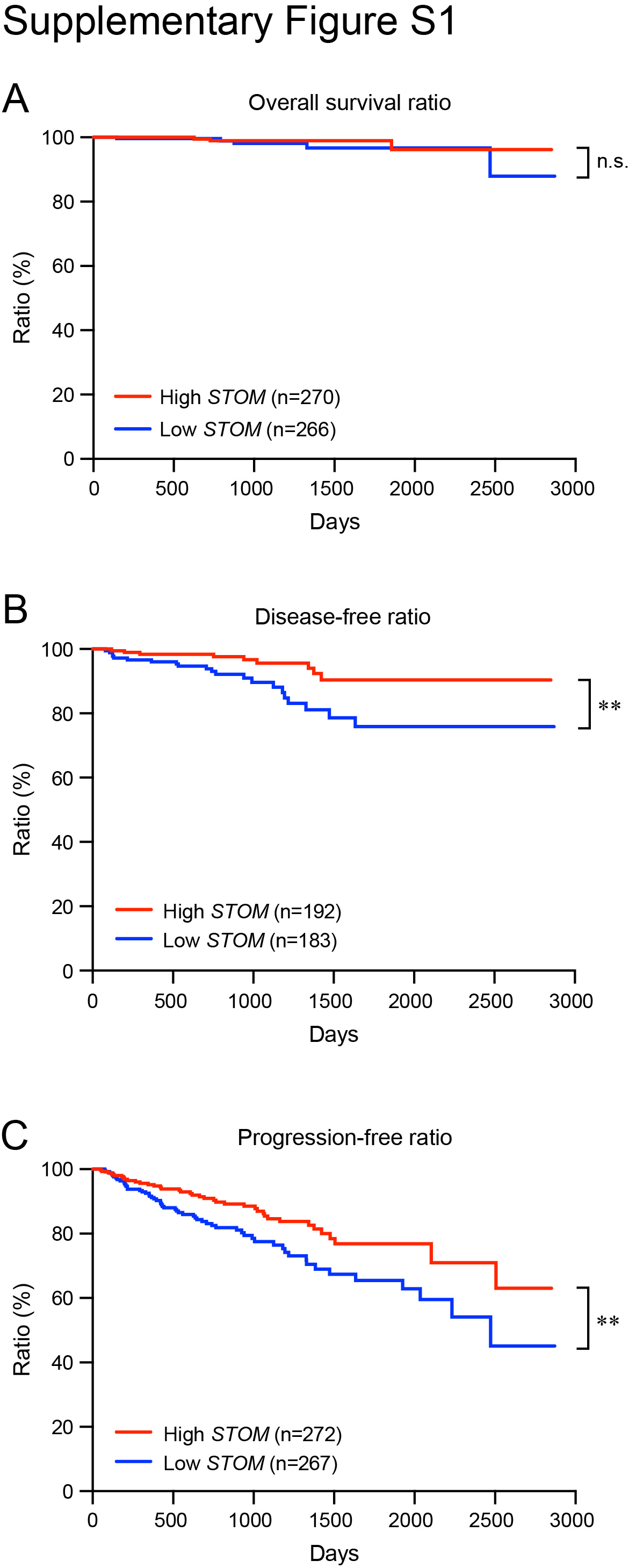

Supplement: Supplementary file 1 — Figure S1. Prognosis of prostate cancer patients with high or low stomatin expression. [file CAM4-13-e70276-s007.jpg]

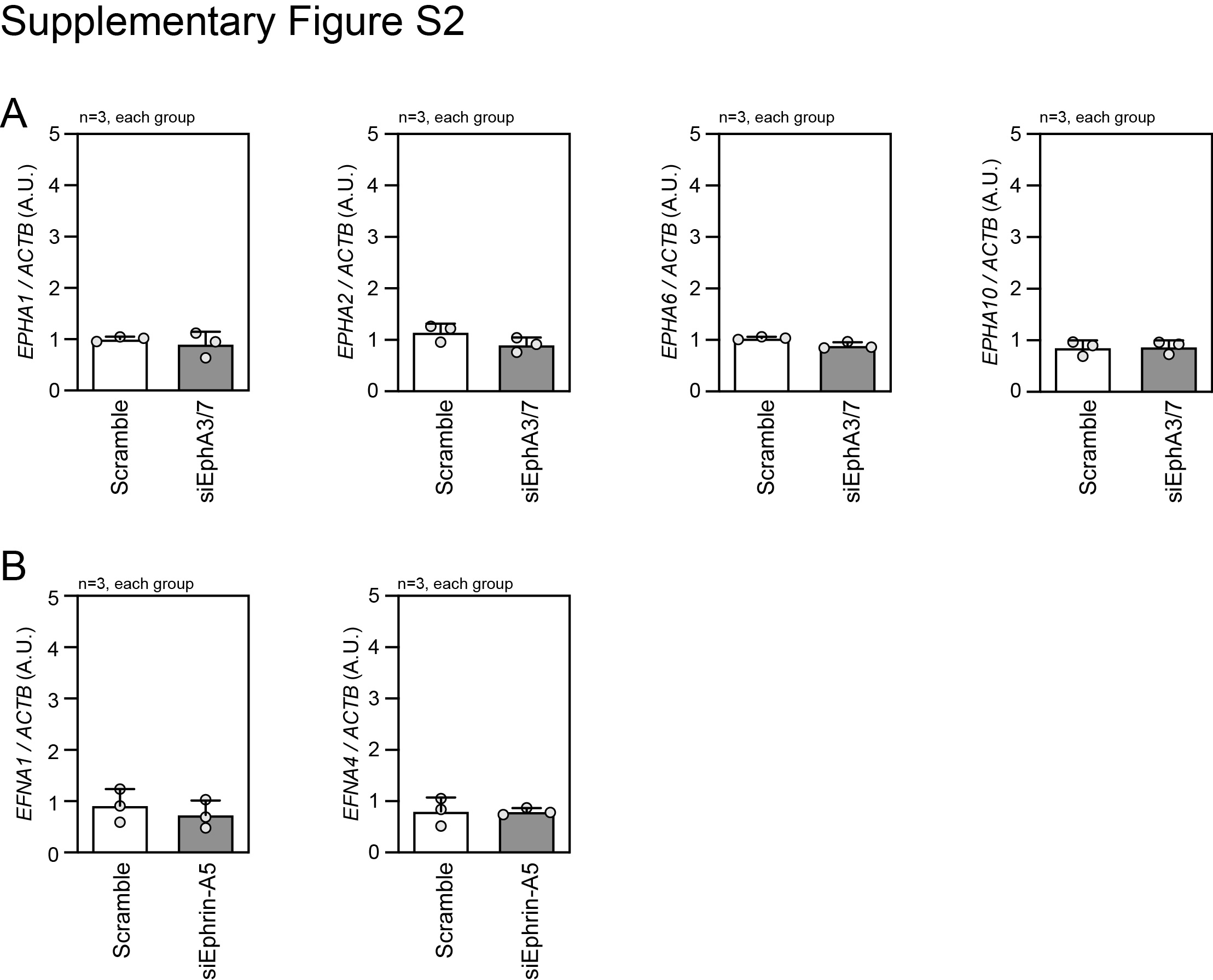

Supplement: Supplementary file 2 — Figure S2. The qPCR analysis for the expression of several EPHA and EFNA genes. [file CAM4-13-e70276-s001.jpg]

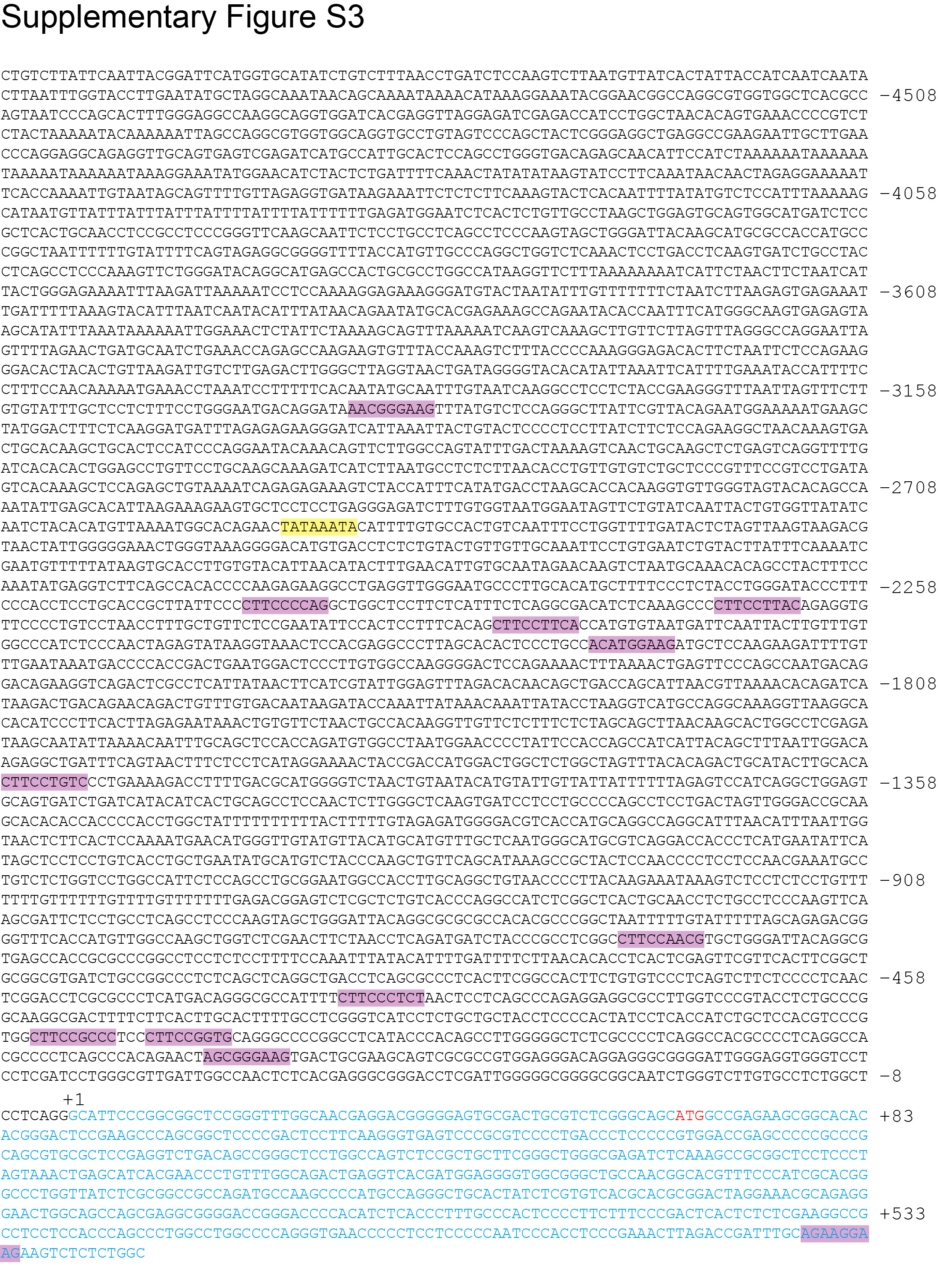

Supplement: Supplementary file 3 — Figure S3. The sequence of the STOM gene promoter region. [file CAM4-13-e70276-s005.jpg]
